# Retrospective analysis of digital health technology effectiveness in cardiac rehabilitation

**DOI:** 10.6026/973206300214138

**Published:** 2025-11-15

**Authors:** Vignesh Krishna Hariharan, Vishnu Poovathinkal Rajan, Sorabh Sharma, Shanmukha Koppolu, Shruthi Alekere Eshu, Nutheti Pavani

**Affiliations:** 1 Department of General Medicine, East Bourne District General Hospital, East Sussex NHS trust, Somerset, United Kingdom; 2 Department of General Medicine, ESIC Hospital, Udyogamandal, Ernakulam, Kerala, India; 3 Department of Medicine, University of Arizona, Arizona, USA; 4 Department of Trauma and Orthopaedics, Barts NHS Trust, London, England, United Kingdom; 5 Department of Medicine, Mandya Institute of Medical Science, Karnataka, India; 6 Department of General Medicine, Madras Medical College, India

**Keywords:** Cardiac rehabilitation, digital health technologies, telemedicine, wearable devices, remote monitoring, patient adherence, cardiovascular risk reduction

## Abstract

Digital health technologies have enhanced cardiac rehabilitation by enabling remote monitoring, personalized feedback and increased patient engagement. This retrospective study analyzed four years of medical records from patients using digital platforms, wearables and teleconsultations. Those using digital tools showed higher adherence, better functional gains and improved risk factor control than those in traditional care. Remote monitoring allowed for earlier detection of clinical changes and timely treatment adjustments, reducing hospital readmissions. Data highlight digital health's potential to expand access and improve long-term outcomes in cardiac care.

## Background:

Cardiac rehabilitation is a cornerstone of secondary prevention among cardiovascular disease patients, intended to restore functional ability, reduce the risk of recurrence and improve quality of life via organized exercise, education and risk factor management. Despite its proven merits, utilization and compliance rates to traditional, center-based care are suboptimal due to considerations such as travel distance, shortage of specialized centers, availability problems and mobility limitations following cardiac event recovery [1]. These issues have prompted the exploration of alternative delivery models with the potential to increase reach and continuity of care beyond traditional venues. Digital health technologies like wearable devices, mobile health (mHealth) applications, remote monitoring systems and teleconsultation platforms have been surfacing as successful tools to bridge this gap [2]. These applications ensure continuous physiological data acquisition, real-time feedback and personalized guidance, allowing patients to engage in rehabilitation at home while maintaining clinical monitoring. Some of the most popular modalities include remote electrocardiogram monitoring, heart rate monitoring, activity tracking and blood pressure monitoring. Also, digital platforms facilitate bi-directional communication between patients and healthcare teams to maximize motivation and compliance with recommended regimens [3]. Evidence from studies and implementation trials reveals that technology-based cardiac rehabilitation is equally effective and in certain situations better than, conventional models of achievement when it comes to clinical outcomes like improved exercise tolerance, risk factor control and quality-of-life indicators. These advantages are particularly evident in groups with geographic, socioeconomic, or mobility limitations. In addition, the COVID-19 pandemic has speeded up the use of telemedicine-based cardiac rehabilitation, making it more relevant and practicable in ensuring unimpeded care during the disruption of healthcare services [4]. Therefore, it is of interest to describe the effectiveness of digital health technologies in cardiac rehabilitation through a retrospective analysis, focusing on functional outcomes, adherence patterns, cardiovascular risk factor management and hospital readmission rates.

## Materials and Methods:

This retrospective analysis was conducted at a tertiary cardiac care center offering both conventional and digitally integrated cardiac rehabilitation programs. Medical records from January 2019 to December 2022 were reviewed to evaluate the impact of digital health technologies on patient outcomes. All adult patients (≥18 years) who had completed at least 12 weeks of cardiac rehabilitation following acute coronary syndrome, percutaneous coronary intervention, coronary artery bypass grafting, or stable chronic coronary artery disease were eligible for inclusion. Two cohorts were identified: (1) patients enrolled in a digitally integrated program combining wearable monitoring devices, mobile health applications and scheduled teleconsultations; and (2) patients participating in standard center-based rehabilitation without digital components. Inclusion criteria consisted of documented baseline and follow-up assessments of functional capacity, cardiovascular risk factors and program adherence. Patients were excluded if they had incomplete records, discontinued rehabilitation before four weeks, or had concurrent conditions that precluded exercise participation, such as severe orthopedic or neurological disorders.

Data were extracted using a standardized proforma and included demographic variables (age, sex, occupation), clinical history (cardiovascular diagnosis, comorbidities such as hypertension, diabetes and dyslipidemia) and rehabilitation program type. Functional capacity was assessed using six-minute walk test (6MWT) distance and peak metabolic equivalents (METs) obtained from exercise stress tests. Cardiovascular risk factor control was evaluated through changes in blood pressure, resting heart rate, LDL cholesterol and HbA1c levels in diabetic patients. Adherence was calculated as the percentage of prescribed rehabilitation sessions attended or completed, based on program logs. Hospital readmissions for cardiovascular causes within six months of program completion were recorded. For the digital cohort, additional variables included average daily step counts, frequency of remote monitoring data uploads and number of teleconsultations attended. The primary outcome was change in functional capacity (6MWT distance and peak METs) from baseline to completion. Secondary outcomes included adherence rates, changes in cardiovascular risk factor profiles and hospital readmission rates. Continuous variables were expressed as mean ± standard deviation or median with interquartile range depending on distribution and compared between groups using independent t-tests or Mann-Whitney U tests. Categorical variables were expressed as frequencies and percentages, with group comparisons using Chi-square or Fisher's exact tests. A p-value of <0.05 was considered statistically significant.

## Results:

A total of 368 patient records met the eligibility criteria, with 182 individuals enrolled in the digitally integrated cardiac rehabilitation program and 186 in the standard center-based program. Both groups were comparable at baseline in terms of age, sex distribution and prevalence of major cardiovascular risk factors, ensuring a balanced comparison. Patients in the digital program achieved significantly higher adherence rates, greater improvements in functional capacity and more favorable changes in cardiovascular risk factor profiles. Remote monitoring and digital engagement were associated with a lower incidence of cardiovascular-related hospital readmissions within six months of program completion. Participants in the digital program demonstrated more consistent physical activity levels, as indicated by wearable device step count data and attended a higher number of scheduled teleconsultations. Patient satisfaction ratings were higher in the digital group, reflecting perceived convenience, continuous feedback and greater accessibility. There were no significant differences in adverse event rates between groups, indicating that the integration of digital technologies did not compromise safety. Multivariate analysis identified digital program participation, higher baseline motivation scores and greater session adherence as independent predictors of functional improvement.

Table 1 (see PDF) demonstrates that the two groups were comparable in demographic and clinical characteristics at baseline, supporting a fair outcome comparison. Table 2 (see PDF) shows that the digital program resulted in significantly greater improvements in six-minute walk test (6MWT) distance and peak METs compared to the standard program. Table 3 (see PDF) demonstrates that adherence rates were significantly higher among digital program participants compared to the standard program. Table 4 (see PDF) shows that the digital program achieved greater reductions in systolic and diastolic blood pressure, LDL cholesterol and resting heart rate. Table 5 (see PDF) demonstrates that the digital program was associated with significantly fewer cardiovascular-related hospital readmissions. Table 6 (see PDF) shows that digital program participants consistently engaged with remote monitoring tools, as indicated by wearable data uploads and teleconsultation attendance. Table 7 (see PDF) demonstrates that patient satisfaction with the rehabilitation process was higher among digital program participants. Table 8 (see PDF) shows that adverse event rates were low and comparable between the two programs, indicating no compromise in safety with digital integration. Table 9 (see PDF) demonstrates that digital program participants had fewer emergency department visits and unscheduled cardiology consultations within six months of completion. Table 10 (see PDF) shows that participation in the digital program, higher baseline motivation scores and greater adherence rates were independent predictors of improved functional capacity. Table 1 (see PDF) presents the baseline demographic and clinical characteristics, showing that both groups were comparable in age, sex distribution and prevalence of major cardiovascular risk factors, ensuring a balanced outcome comparison. Table 2 (see PDF) shows that the digital program led to significantly greater improvements in six-minute walk test distance and peak METs compared to the standard program, reflecting superior gains in functional capacity. Table 3 (see PDF) demonstrates that adherence rates were markedly higher among participants in the digital program, with a greater proportion achieving ≥80% attendance compared to the standard program. Table 4 (see PDF) presents the changes in cardiovascular risk factors, indicating that the digital program achieved larger reductions in blood pressure, LDL cholesterol and resting heart rate. Table 5 (see PDF) shows that cardiovascular-related hospital readmission rates within six months were significantly lower in the digital program compared to standard rehabilitation. Table 6 (see PDF) demonstrates high engagement levels among digital program participants, with strong compliance in wearable device data uploads, consistent step counts and frequent teleconsultation attendance. Table 7 (see PDF) presents patient-reported satisfaction scores, showing higher ratings for overall satisfaction, convenience and perceived health improvement in the digital group. Table 8 (see PDF) shows that adverse event rates were low and comparable between groups, indicating no compromise in safety with the integration of digital technologies. Table 9 (see PDF) demonstrates that digital program participants had fewer emergency department visits and unscheduled cardiology consultations within six months after program completion. Table 10 (see PDF) presents the independent predictors of functional improvement, identifying digital program participation, high baseline motivation and strong adherence as significant positive factors.

## Discussion:

This retrospective analysis assessed the effectiveness of digital health technologies integrated into cardiac rehabilitation programs, comparing them with conventional center-based models. The findings consistently demonstrated that the incorporation of wearable monitoring devices, mobile health applications and teleconsultations led to superior outcomes in functional capacity, adherence, cardiovascular risk factor control and healthcare utilization without compromising safety. These findings add to the increasing body of evidence for the contribution of technology-based interventions to increasing the reach, activity and clinical outcomes of rehabilitation for cardiovascular disease patients [5]. The similar baseline population characteristics guaranteed variation in outcome resulted from the type of rehabilitation rather than resulting from intrinsic variability. The improved functional capacity seen with the digitally integrated group is consistent with synergistic effect between individualized feedback, real-time monitoring and enhanced patient interaction. The capacity to monitor activity levels, track intensity of exercise and remotely adjust targets could have facilitated such gains in performance. Higher physical activity compliance in leisure time, as monitored by wearables, is an essential predictor of maintenance of such gains in the longer term [6]. Adherence was the strongest discriminator between groups and was associated with almost doubling the attendance and participation in the online program. This can be explained by flexible time, convenience of exercising at home and consistent digital communication that might have overcome the conventional impediments like lack of transport, limited time and work schedule. Good compliance is highly attributed to further improvement in exercise capacity and cardiovascular risk profiles and that technology-assisted participation is a cause of clinical success [7]. Control of cardiovascular risk factors was better in the digital program, with more lowering of blood pressure, LDL cholesterol and resting heart rate. This can be partly explained by increased patient-provider contact, with the potential for timely drug titration and lifestyle advice. On-going self-monitoring can sustain behavior change, enhance diet compliance and keep patients engaged, all of which contribute to risk factor control [8]. The reduced hospital readmission rate in the digital group presents a potential benefit to the health system, both through lowering costs of acute care and enhancing the quality of life for the patient. Preventative measures enabling prevention of hospitalization are enabled through early detection of clinical deterioration via remote monitoring. Similarly, the reduced emergency visit and unplanned cardiology consultation rates underscore the value of active surveillance and timely follow-up in the prevention of symptom escalation [9]. Patient satisfaction was significantly higher in the digital program, a surrogate marker of ease, convenience and accessibility and a sense of continuous clinical support. All these dimensions are significant for long-term program sustainability, as enhanced patient experience has the potential to boost compliance with rehabilitation guidelines and encourage such programs among peers. Notably, the incorporation of technology did not result in higher rates of adverse events, which provides evidence supporting remote and hybrid models of rehabilitation safety when applied with due safeguards [10].

Multivariate analysis validated that digital program participation; high baseline motivation and high adherence were independent predictors of increased functional improvement. This illustrates the multifactorial causality of rehabilitation success through the integration of technology-facilitated infrastructure with patient-specific behavioral influences. Enhancing the effectiveness of digital interventions with identification and development of motivational drivers may be a future strategy [11]. These findings suggest that digital health technologies can serve as a robust adjunct or even an alternative to traditional cardiac rehabilitation, especially for patients with logistical barriers to attending in-person sessions. Hybrid models blending center-based initiation with remote follow-up may be particularly effective, offering initial supervised training followed by sustained home-based engagement [12]. From a policy and implementation standpoint, scaling such digital programs will require investment in technology platforms, training of healthcare professionals in digital engagement and ensuring equitable access for patients across different socioeconomic and geographic contexts. Special consideration must be given to older adults or those with limited digital literacy to avoid widening disparities in care. American Heart Association science advisory was assembled to guide the development and implementations of digital cardiac rehabilitation interventions that can be translated effectively into clinical care, improve health outcomes, and promote health equity. This advisory thus describes the individual digital components that can be delivered in isolation or as part of a larger cardiac rehabilitation telehealth program and highlights challenges and future directions for digital technology generally and when used in cardiac rehabilitation specifically [13].

Limitations of this study include its retrospective design, which is inherently subject to potential selection bias and reliance on medical record documentation, which may omit some patient-reported outcomes. The single-center setting may limit generalizability and longer follow-up is needed to assess the durability of benefits beyond the six-month period evaluated. Future prospective, multicenter studies with cost-effectiveness analyses would further clarify the role of digital health technologies in cardiac rehabilitation. Integrating digital health technologies into cardiac rehabilitation programs enhances functional recovery, improves adherence, optimizes cardiovascular risk factor control and reduces healthcare utilization without compromising safety. This approach offers a scalable, patient-centered strategy to modernize secondary prevention in cardiovascular care and improve long-term patient outcomes.

## Conclusion:

Digital health technologies integrated into cardiac rehabilitation programs significantly improve functional capacity, adherence, cardiovascular risk factor control and healthcare utilization outcomes without compromising safety. Wider adoption of such technology-enabled models has the potential to enhance access, sustain patient engagement and optimize long-term cardiovascular health in diverse populations.
